# Dopamine transporter silencing in the rat: systems-level alterations in striato-cerebellar and prefrontal-midbrain circuits

**DOI:** 10.1038/s41380-022-01471-4

**Published:** 2022-03-04

**Authors:** Jonathan R. Reinwald, Natalia Gass, Anne S. Mallien, Alexander Sartorius, Robert Becker, Markus Sack, Claudia Falfan-Melgoza, Christian Clemm von Hohenberg, Damiana Leo, Natascha Pfeiffer, Anthonieke Middelman, Andreas Meyer-Lindenberg, Judith R. Homberg, Wolfgang Weber-Fahr, Peter Gass

**Affiliations:** 1 Research Group Translational Imaging, Department of Neuroimaging, Central Institute of Mental Health, Medical Faculty Mannheim, University of Heidelberg, Heidelberg, Germany; 2 Department of Psychiatry and Psychotherapy, Central Institute of Mental Health, Medical Faculty Mannheim, University of Heidelberg, Heidelberg, Germany; 3 Research Group Systems Neuroscience and Mental Health, Department of Psychiatry and Psychotherapy, University Medical Center Mainz, Johannes Gutenberg University, Mainz, Germany; 4 Research Group Animal Models in Psychiatry, Central Institute of Mental Health, Medical Faculty Mannheim, University of Heidelberg, Heidelberg, Germany; 5 Center for Innovative Psychiatry and Psychotherapy Research, Central Institute of Mental Health, Medical Faculty Mannheim, University of Heidelberg, Heidelberg, Germany; 6 Department of Neurosciences, University of Mons, Mons, Belgium; 7 Centre for Neuroscience, Department of Cognitive Neuroscience, Donders Institute for Brain, Cognition, and Behaviour, Radboud University Nijmegen Medical Centre, Nijmegen, the Netherlands

**Keywords:** Neuroscience, Genetics, ADHD, Schizophrenia, Bipolar disorder

## Abstract

Silencing of dopamine transporter (DAT), a main controlling factor of dopaminergic signaling, results in biochemical and behavioral features characteristic for neuropsychiatric diseases with presumed hyperdopaminergia including schizophrenia, attention deficit hyperactivity disorder (ADHD), bipolar disorder, and obsessive-compulsive disorder (OCD). Investigation of DAT silencing thus provides a transdiagnostic approach towards a systems-level understanding of common underlying pathways. Using a high-field multimodal imaging approach and a highly sensitive cryogenic coil, we integrated structural, functional and metabolic investigations in tandem with behavioral assessments on a newly developed preclinical rat model, comparing DAT homozygous knockout (DAT-KO, *N* = 14), heterozygous knockout (*N* = 8) and wild-type male rats (*N* = 14). We identified spatially distributed structural and functional brain alterations encompassing motor, limbic and associative loops that demonstrated strong behavioral relevance and were highly consistent across imaging modalities. DAT-KO rats manifested pronounced volume loss in the dorsal striatum, negatively correlating with cerebellar volume increase. These alterations were associated with hyperlocomotion, repetitive behavior and loss of efficient functional small-world organization. Further, prefrontal and midbrain regions manifested opposite changes in functional connectivity and local network topology. These prefrontal disturbances were corroborated by elevated myo-inositol levels and increased volume. To conclude, our imaging genetics approach provides multimodal evidence for prefrontal-midbrain decoupling and striato-cerebellar neuroplastic compensation as two key features of constitutive DAT blockade, proposing them as transdiagnostic mechanisms of hyperdopaminergia. Thus, our study connects developmental DAT blockade to systems-level brain changes, underlying impaired action inhibition control and resulting in motor hyperactivity and compulsive-like features relevant for ADHD, schizophrenia and OCD.

## Introduction

Disturbances in the dopaminergic system are crucially involved in many neuropsychiatric disorders including schizophrenia, attention-deficit-hyperactivity disorder (ADHD), addiction, bipolar disorder, and obsessive-compulsive disorder (OCD), underlying motor impairments, hallucinations, and cognitive and emotional deficits [1, 2]. These disorders are often comorbid and present overlapping symptoms with common etiology [3, 4]. The investigation of how specific disturbances in dopaminergic signaling affect neural circuits on the systems-level can thus reveal common transdiagnostic dopamine-related pathomechanisms, contributing to biologically-based understanding according to the Research Domain Criteria framework [5].

One of the critical brain systems in dopaminergic signaling are basal ganglia, constituting a part of motor, associative and limbic loops and regulating locomotor, cognitive and reward-related behavior [6]. Interactions between basal ganglia and prefrontal cortex mediate inhibition of ongoing actions - a function crucially important for cognitive control and flexibility [7]. Dopaminergic imbalance leads to impaired behavioral inhibition characteristic for ADHD, OCD, and schizophrenia, and expressed as involuntary motor activity, compulsive-like features and habitual rather than goal-directed behavior [8, 9]. The dopamine transporter (DAT), a transmembrane protein that removes dopamine from extracellular space, serves as the main controlling factor of dopaminergic neurotransmission [10]. Impaired dopamine clearance due to DAT deficiency results in prolonged dopamine surge with consequent loss of informational value from dopamine signals. Mutations or polymorphisms in the DAT gene *SLC6A3* contribute to ADHD [11], OCD [12], bipolar disorder [13] and schizophrenia [14]. Therefore, systematic investigations of DAT dysfunction provide a transdiagnostic approach towards better understanding of the common underlying mechanistic pathways across disorders. However, such investigations remain challenging in humans, as various factors (environmental influences, medication history, heterogeneous samples) contribute to neural changes, often interacting in an unknown way. Animal models offer an opportunity to investigate the effects of specific dopaminergic alterations in a controlled environment overcoming some limitations of human studies.

In this work, we used a newly developed DAT knockout (DAT-KO) rat model [15]. Compared with the mouse model [16], it offers closer physiological similarity to humans and more precise translatability [15]. DAT-KO rats display features characteristic for ADHD, schizophrenia, OCD, and additionally anhedonia [17]. Compulsive OCD-like features include reduced spontaneous alternation in Y-maze [15], increased marble hiding [18], inflexibility in dynamic reward tasks, and compulsive destroying of objects [17]. ADHD aspects are represented by hyperactivity, antihyperkinetic responses to psychostimulants [15], and impaired working memory [19]. Schizophrenia-like features include compromised sensorimotor gating and functional hyperdopaminergia [15]. These disorders are etiologically related to impaired basal ganglia function [20, 21]. Similarly, the DAT-KO model exhibits basal ganglia abnormalities including hyperdopaminergia, reduced brain-derived neurotrophic factor (BDNF) and postsynaptic density protein 95 (PSD-95) levels in the striatum [15]. Thus, the DAT-KO model reflects not only a transdiagnostic symptomatology, but also suggests a shared pathomechanism. However, profound understanding of circuit alterations induced by impaired DAT function is lacking.

In order to investigate the systems-level effects of DAT blockade, we combined structural and functional magnetic resonance imaging (fMRI) with MR-spectroscopy and behavioral testing. Our multimodal approach allows a simultaneous analysis of brain functional, structural and metabolic changes in tandem with behavior, thus revealing direct neuroimaging correlates of behavioral deviances. We have focused our functional analysis on the motor, limbic and associative loops with striatum at their core, as (a) dopaminergic alterations within these circuits lead to disturbances in motor, affective and cognitive domains, characteristic for ADHD, OCD and schizophrenia; (b) striatum exhibited the most robust structural changes in the first part of our analysis.

## Materials and methods

### Animals and experimental design

Three groups of male rats (*N* = 14 DAT-KO, *N* = 8 DAT heterozygous (DAT-HET), *N* = 14 wild-type (WT); Wistar-Han background) were bred in the Radboud University (Nijmegen, the Netherlands) and transported to the Central Institute of Mental Health (Mannheim, Germany) at the age of 3–4 weeks. The animals were tested for explorative and locomotor behavior in the open field test (OFT) and for sensorimotor gating in the prepulse inhibition (PPI) test, as previously described [22], at age of 11 weeks on two consecutive days. In the OFT, an individual rat was placed for 30 min into an illuminated unfamiliar arena and locomotor activity was recorded. Movement (%), distance moved and center time were analyzed using EthoVision® XT (Noldus, Wageningen, Netherlands). In the PPI test, prepulses were presented 100 ms before the startle stimulation with four different intensities (72, 76, 80, 84 dB). Each trial type was presented 10 times and PPI was calculated as the individual percentage decrease of the startle response magnitude in trials with preceding prepulse (for details, see *Supplement*). MRI measurements were conducted 7 days later (Fig. S1). To compose the daily schedules of MRI measurements, we used randomization according to group (WT/DAT-HET/DAT-KO) and time of the day. The experiments were performed according to the regulations covering animal experimentation within the European Union (European Communities Council Directive 2010/69/EU) and within the German Animal Welfare Act, and were approved by the German animal welfare authorities (Regierungspräsidium Karlsruhe, 35-9185-81-G-143-19).

### MRI acquisition

The MRI experiments were conducted at a 9.4 T MR-scanner (Bruker BioSpec, Ettlingen, Germany) using a cryogenically cooled rat brain coil (four-channel phased array MRI CryoProbe^TM^ with a room-temperature volume transmit coil, Bruker BioSpec) which, compared to standard room temperature coils, provides a signal-to-noise ratio increased by 2.4. Anesthesia was performed as previously described [23, 24] combining 0.5% isoflurane (Baxter Deutschland GmbH, Unterschleissheim, Germany) with medetomidine (0.06 mg/kg/h, s.c.; Domitor, Janssen-Cilag, Neuss). We monitored sedation depth via recording respiratory and cardiac signals (details in *Supplement*). Isoflurane dose in four animals was slightly reduced to keep an appropriate sedation depth. Importantly, we found no group differences for isoflurane levels, respiration and heart rate (p > 0.05, one-way ANOVA), suggesting comparable anesthesia depth (Fig. S2). Nevertheless, isoflurane dose was included as a covariate in fMRI and MR-spectroscopy analyses.

MRI acquisition protocol included: (a) high-resolution 3D scan (T2-weighted rapid-acquisition-with-refocused-echoes sequence, repetition time/echo time TR/TE 1200/50 ms, voxel dimensions 0.15 ×0.15 ×0.288 mm^3^); (b) MR-spectroscopy (point-resolved-spectroscopy sequence, 12-µl-volume (3 ×2 x 2 mm³) voxel in the prelimbic-cingulate cortex, TR/TE 4000/10 ms); (c) FieldMap (TR-short/TE-long/TE 20/1.7/5.7 ms); (d) resting-state fMRI (rsfMRI) (T2*-weighted echo-planar-imaging-free-induction-decay sequence, TR/TE 1500/17.5 ms, voxel in-plane dimension 0.365 mm, 350 acquisitions). For sequences’ details see *Supplement*. The preprocessing steps for all imaging modalities are illustrated in Fig. S3.

### MR-spectroscopy

MR-spectroscopy focused on the prelimbic-cingulate cortex as a region with crucial transdiagnostic relevance for cognitive top-down control and flexibility. The volume was placed to maximize prelimbic-cingulate coverage, while excluding white matter, cerebrospinal fluid and neighboring regions (e.g. striatum) to avoid partial volume effects confounding the signal of the target region. In the sagittal plane the voxel was placed between the anterior edge of the corpus callosum and the border between olfactory bulb and forebrain with an angulation parallel to the borderline. To minimize chemical shift displacement artifacts, volume-selective radiofrequency pulses were shifted by −2 ppm into the range between glutamate and myo-inositol reference lines. An additional one-shot unsuppressed water spectrum was acquired with no frequency shift, which was used for eddy-current correction and water-scaling. Spectra were quantified by LCModel [25] using a basis dataset obtained from calibrated metabolite solutions at the same MR-scanner with the same sequence. Metabolite concentration values were referenced to the unsuppressed water signal, assuming mean water concentration of 46.106 mol/L [26], and corrected for relaxation effects (T1/T2_met_ 1500/300 ms, T2_water_ 45 ms) [27]. Low quality spectra were excluded; only metabolites with a Cramer-Rao-Lower-Bound of <20% were taken into account.

### Deformation-based morphometry (DBM)

DBM analysis allows for a whole-brain voxel-based investigation without a priori assumptions on brain parcellation, thereby detecting volumetric alterations in specific clusters. DBM analysis was performed using SPM12 (https://www.fil.ion.ucl.ac.uk/spm/software/spm12/) and in-house MATLAB scripts (MATLAB R2020a; MathWorks Inc., USA). Preprocessing included brain extraction, bias correction, coregistration, segmentation based on tissue probability maps from the SIGMA rat brain atlas [28] and normalization using Diffeomorphic Anatomical Registration Through Exponentiated Lie Algebra (DARTEL) tool [29] to obtain animal-specific Jacobian determinant maps representing transformation of individual animal maps to the average-shaped template.

To assess differences in relative brain volumes, Jacobian deformation maps were tested in second-level analysis with total brain volume (TBV) as a covariate (SPM12). SPM-based cluster-correction with a cluster-defining primary threshold of *p* < 0.01 was used for multiple comparison correction. To investigate the robustness of our findings, we additionally applied a stringent voxel-wise family-wise error (FWE) rate correction on threshold-free cluster-enhanced Jacobian maps.

To further investigate associations between structural changes and behavioral and functional features (e.g. graph metrics) in the DAT-KO group, we quantified regional volumetric changes applying region of interest (ROI)-based analysis. The SIGMA atlas was used for parcellation [28] (Fig. S4). ROI volumes were calculated as a mean value, based on individual Jacobian maps. To account for TBV differences, regional volumes were transformed into percentages of individual TBV and were used for group comparison and correlation analyses. For details, see *Supplement*.

### RsfMRI preprocessing

Image preprocessing was performed as in our previous studies [30, 31] and included the following steps: (a) correction for magnetic field inhomogeneities and movement (“realign & unwarp”, SPM12); (b) regressing respiratory and cardiac signals (Aztec software) [32]; (c) slice-timing correction (SPM12); (d) coregistration and spatial normalization to SIGMA atlas template [28] using DARTEL-based flow fields (obtained from the 3D image coregistration and normalization) (SPM12); (e) regressing movement parameters and cerebrospinal fluid signal (FSL, version 5 http://www.fmrib.ox.ac.uk/fsl); (f) identification of motion-affected frames (Fig. S2) based on DVARS decomposition [33] and removal, based on *p* values instead of an arbitrary threshold, with subsequent scrubbing (spline-curve interpolation) [34]; (g) band-pass filtering (0.01–0.1 Hz) (Analysis of Functional NeuroImages, version 2) [35].

Groups manifested no differences in mean motion and number of motion-affected frames. For all animals, motion affected less than 10% of all timeframes and was on a relatively low level (Fig. S2).

### Network-based statistic (NBS)

NBS, a non-parametric cluster-based method identifying potential connected structure and controlling multiple comparisons [36], was used to assess functional connectivity (FC) within a network encompassing 26 key regions of associative, limbic and motor circuits (Fig. 4). Mean regional time-courses were extracted and Pearson’s correlation coefficients between the time-courses were computed, resulting in individual networks, with nodes representing regions and edge weights correlation coefficients. The statistical model was specified in terms of general linear model with F-tests performed for each edge. Two primary thresholds (stringent p_pt_ < 0.01 (F_26_ = 7.72 to F_20_ = 8.09), more lenient p_pt_ < 0.05 (F_26_ = 4.23 to F_20_ = 4.35)) were separately used to discard sub-threshold edges. The contiguous surviving edges were defined as a cluster. The cluster intensity was compared with the maximum intensity resulting from 5.000 random permutations (p_NBS_ < 0.05). NBS was performed on connectivity matrices already corrected for differences in isoflurane levels using multivariate analysis of covariance.

### Graph-theoretical analysis

Graph-theoretical analysis is a complementary method assessing distinct brain network features which cannot be quantified with NBS. Modeling the brain as a large complex network, graph analysis quantifies whole-brain information exchange with global metrics and characterizes the influence of individual brain regions in network communication with local metrics. Graph analysis was done as previously [31], using 110 unihemispheric ROIs from SIGMA atlas [28] (Fig. S4), excluding olfactory bulb, cerebellum and brainstem as not covered by rsfMRI sequence. Pearson’s correlation matrices calculated from mean regional time-courses (normalized by maximum weights) were used to compute network metrics (Brain Connectivity Toolbox, version 2016-01-16) [37]. Networks were binarized by retaining 5–40% of the strongest edges (1% step), and normalized, using random graphs preserving node number, degree distribution, and connectedness [38]. First, each graph metric was calculated for every threshold; afterwards, mean metric value across thresholds was used to identify systematic effects that are not dependent on a specific threshold. To explore changes in global topology, we calculated small world propensity (SWP), global efficiency, mean local efficiency and robustness against targeted and random attacks. Degree, local clustering coefficient and participation index were assessed to explore regional network characteristics (see definitions in *Supplement*). Of note, group differences for graph metrics demonstrated stability over the range of selected thresholds, as exemplarily illustrated for global efficiency in Fig. S5.

### Statistics

Non-parametric one-way ANOVA using permutation tests (MATLAB function *randanova1*, 10.000 permutations) was applied to estimate *p* values for group differences in behavioral performance, since the O’Brien test for homogeneity of variances revealed unequal variances between the groups. One-way ANOVA with Tukey-Kramer post hoc tests for multiple comparison correction was used to investigate differences in structural, graph analytical and spectroscopy metrics between the three groups. Pearson’s correlation analyses were computed to investigate associations between graph metrics, regional brain volume, myo-inositol levels and behavioral traits specifically in the DAT-KO group. For graph metrics and spectroscopy, one-way ANOVA and partial correlation analyses included isoflurane as a covariate.

## Results

### DAT-KO rats display hyperactivity, thigmotaxis and sensory gating deficits

**Fig. 1:**
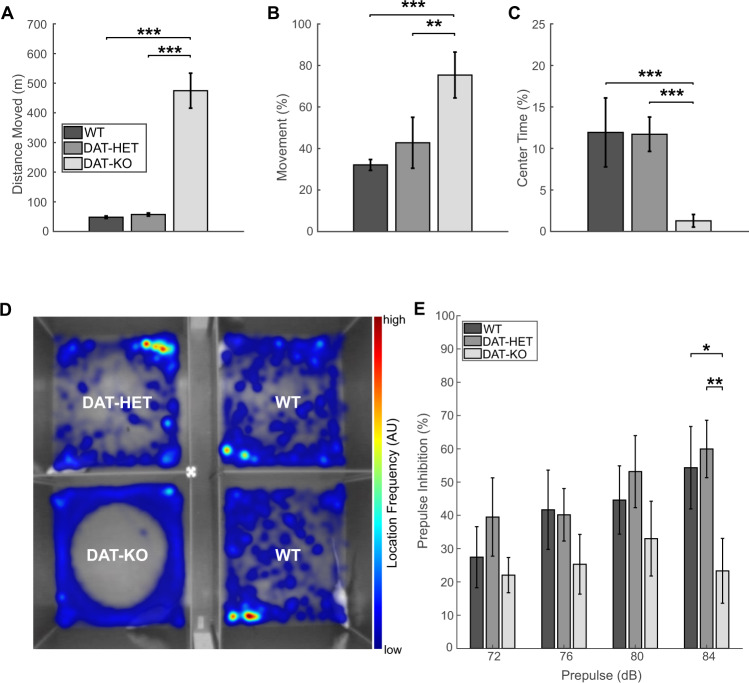
Behavioral alterations in DAT-KO, DAT-HET and WT rats. **A** Mean values (±SEM) of the total distance moved in the open field test. **B** Mean values (± SEM) of percentual time spent in motion in the open field test. **C** Mean values (± SEM) of time spent in the center in the open field test. **D** Typical movement patterns in the open field test illustratively presented for *N* = 4 animals (*N* = 2 WT rats), with DAT-KO rats demonstrating a repetitive circling pattern with center avoidance. **E** Differences in prepulse inhibition (in percentage from startle, mean values ± SEM) for four sound pressure levels (72, 76, 80, and 84 dB). AU arbitrary units, dB decibel, SEM standard error of the mean; *p < 0.05; **p < 0.01; ***p < 0.001.

In the OFT, as compared to WT and DAT-HET rats, DAT-KO rats demonstrated prominent hyperactivity, indicated by increased total distance moved (F_2,33_ = 151.82, *p* < 0.001 for both) (Fig. 1A) and longer motion time (F_2,33_ = 27.69, *p* < 0.001 and *p* < 0.01, respectively) (Fig. 1B). They also spent significantly less time in the center (F_2,33_ = 17.42, *p* < 0.001 for both) (Fig. 1C) and moved in a repetitive circling pattern along walls, indicating thigmotaxis (Fig. 1D)—a feature so far demonstrated only in DAT-KO mice [39]. DAT-KO rats displayed significantly reduced PPI at 84 dB, compared to WT and DAT-HET rats (F_2,25_ = 9.43, *p* < 0.05 and *p* < 0.01, respectively) (Fig. 1E).

### DBM analysis reveals broad volume changes encompassing motor, associative and limbic loops

**Fig. 2:**
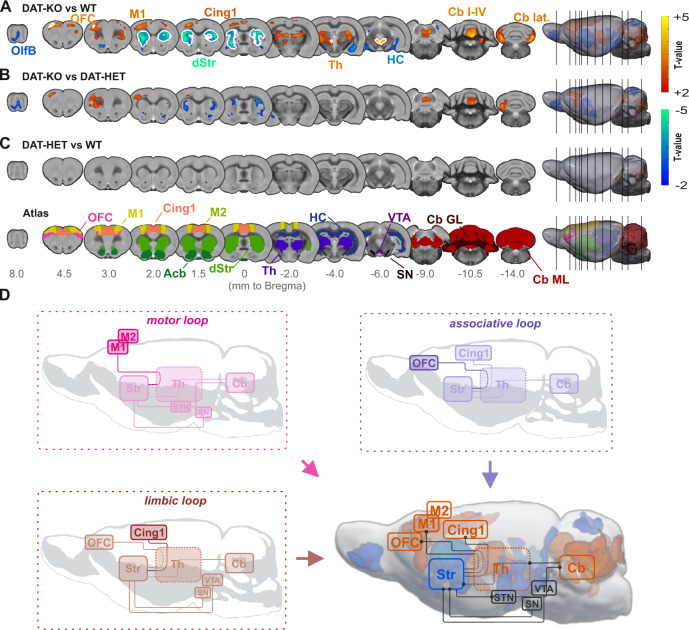
Brain morphology comparison between DAT-KO, DAT-HET and WT rats and illustration of associative, limbic and motor loops affected by volumetric changes in DAT-KO rats. **A** Comparison between DAT-KO and WT rats revealed a significant pattern of decreased relative brain volume in DAT-KO rats (blue scale), with clusters covering predominantly striatum, hippocampus and olfactory bulb. In contrast, relative brain volume in orbitofrontal, cingulate and motor regions as well as in thalamus, midbrain and cerebellum were significantly increased (red scale). **B** Comparison between DAT-KO and DAT-HET rats revealed a comparable, however less pronounced pattern. **C** Comparison between DAT-HET and WT rats detected no significant differences between these groups. **D** Morphological alterations in DAT-KO compared to WT rats demonstrate an overlap with motor, limbic and associative loops (orange—volume gain, blue—volume loss, thresholded at *p* < 0.01, whole-brain analysis). For visualization purposes, all results in (**A–C**) are thresholded at a cluster-defining-threshold level of *p* < 0.01 and only clusters with a size larger than 500 voxels are plotted. White contours signify regions surviving threshold-free cluster enhancement with voxel-wise whole-brain family-wise error correction at *p* < 0.05. Acb accumbens, Cb cerebellum, Cb I-IV cerebellar lobules I-IV, Cb lat. cerebellum, lateral part, Cb GL granular layer of the cerebellum, Cb ML molecular layer of the cerebellum, Cing1 cingulate cortex area 1, dStr dorsal striatum, HC hippocampus, M1 primary motor cortex, M2 secondary motor cortex, OFC orbitofrontal cortex, OlfB olfactory bulb, SN substantia nigra, STN subthalamic nucleus, Th thalamus, VTA ventral tegmental area.

Finding a significant reduction in TBV of DAT-KO rats compared to DAT-HET (*p* < 0.001) and WT rats (*p* < 0.05) (Fig. S6), we included it as a covariate to avoid confounding effects. Whole-brain investigation with a cluster-defining threshold of *p* < 0.01 revealed a highly significant reduction of relative brain volume covering striatum (bilateral cluster, *p* < 0.001, cluster-corrected), hippocampus (bilateral cluster, *p* < 0.001, cluster-corrected) and olfactory bulb (*p* < 0.01, cluster-corrected) in DAT-KO rats (Fig. 2A). In contrast, a relative enlargement occurred in cortical regions, such as orbitofrontal (OFC), cingulate, primary and secondary motor (M1, M2) (*p* < 0.001, cluster-corrected), as well as in thalamus, midbrain and cerebellum (lobules I-IV) (*p* < 0.001, cluster-corrected) (Fig. 2A). Main volume alterations survived threshold-free cluster-enhancement with stringent voxel-wise FWE correction at *p* < 0.05, further underlining the high robustness of our results (illustrated with white contours in Fig. 2A). Whole-brain findings with a more stringent cluster-defining threshold of p < 0.001 are illustrated in Fig. S7. Comparison between DAT-KO and DAT-HET groups yielded similar results, though on a smaller scale: modest enlargement emerged in the cerebellar lobules (*p* < 0.01, cluster-corrected) and frontal cortical regions (OFC and M1, *p* < 0.001, cluster-corrected), while a reduction was found in striatum and olfactory bulb (*p* < 0.01, cluster-corrected) (Fig. 2B). No differences were detected between DAT-HET and WT groups (Fig. 2C). Region-specific investigations of volume differences confirmed whole-brain findings (Fig. S7). Of note, volume changes in DAT-KO rats overlapped with key regions of the motor, associative and limbic loops (Fig. 2D).

### Inter-regional DBM analyses in DAT-KO rats identify anti-correlations in striatal-cerebellar axis

**Fig. 3:**
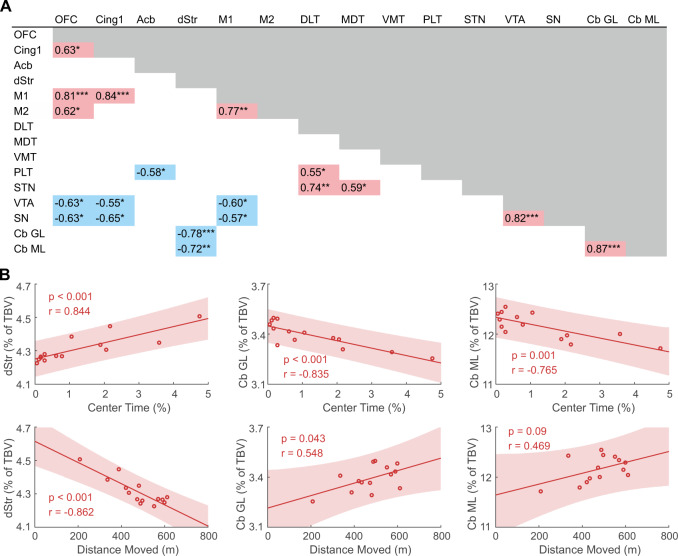
Correlations between relative regional volumes of motor, limbic, and associative loops and between relative striato-cerebellar volumes and behavioral metrics of the open field test in DAT-KO rats. **A** Pearson’s correlation coefficients between relative regional volumes in DAT-KO rats. Negative correlation coefficients are marked in blue, positive correlation coefficients in red. Volume of the dorsal striatum was anti-correlated with cerebellar volume, while orbitofrontal, motor and cingulate volumes were positively correlated with each other and anti-correlated with subcortical volumes of the ventral tegmental area and the substantia nigra. **B** Pearson’s correlation coefficients of the DAT-KO group between regional volume in the striato-cerebellar axis and behavioral metrics in the open field test. Striatal volume showed strong negative correlation with distance traveled and positive with center time, while cerebellar regions displayed an opposite pattern. Cb GL granular layer of the cerebellum, Cb ML molecular layer of the cerebellum, Cing1 cingulate cortex area 1, DLT dorsolateral thalamus, dStr dorsal striatum, M1 primary motor cortex, M2 secondary motor cortex, MDT mediodorsal thalamus, OFC orbitofrontal cortex, PLT posterolateral thalamus, SN substantia nigra, STN subthalamic nucleus, TBV total brain volume, VMT ventromedial thalamus, VTA ventral tegmental area. *p < 0.05; **p < 0.01; ***p < 0.001.

To dissect potential mechanistic links underlying prominent volumetric changes in motor and associative-limbic loops in the DAT-KO group, we investigated inter-regional volume correlations within these circuits. Pointing towards a potential compensatory mechanism within the cerebellar part of the loops, striatal volume strongly anti-correlated with cerebellar volume (granular layer (Cb GL) *r* = −0.78, *p* < 0.001; molecular layer (Cb ML) *r* = −0.72, *p* < 0.01) (Fig. S8), while not displaying significant correlations with other circuit regions (*p* > 0.05). In contrast, cortical volumes positively correlated with each other, while being anti-correlated with volumes of subcortical dopaminergic hub regions (ventral tegmental area (VTA) and substantia nigra (SN)) (Fig. 3A).

### Striato-cerebellar alterations correlate with behavior in DAT-KO rats

Detecting prominent but anti-correlated structural alterations of the striato-cerebellar regions, we investigated their relevance for hyperactivity and thigmotaxis observed in DAT-KO rats. Striatal volume showed strong negative correlation with distance traveled (*r* = −0.862, *p* < 0.001), and positive with center time (*r* = 0.844, *p* < 0.001) (Fig. 3B). On the contrary, cerebellar subregions displayed positive correlations with distance traveled (Cb GL *r* = 0.548 *p* < 0.05), while anti-correlating with center time (Cb GL *r* = −0.835, *p* < 0.001; Cb ML *r* = −0.765, *p* = 0.001) (Fig. 3B).

### RsfMRI analyses demonstrate frontal-midbrain decoupling in DAT-KO rats

**Fig. 4:**
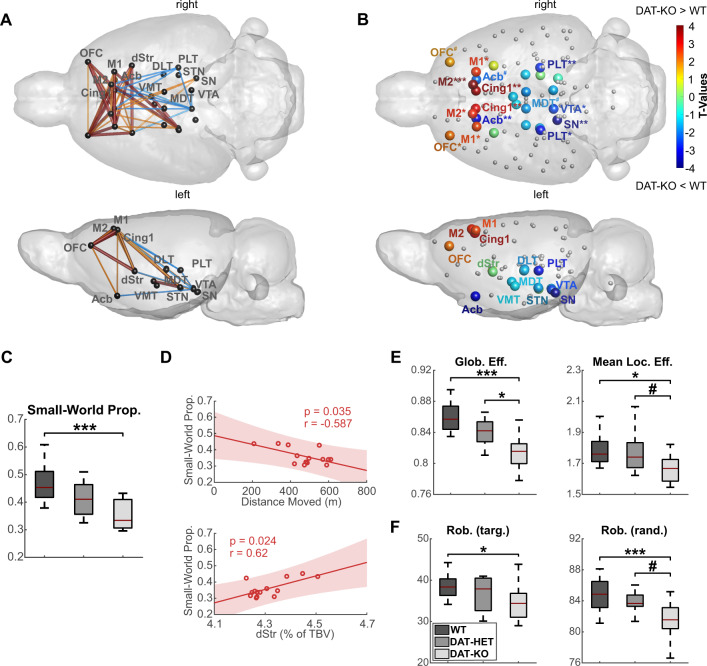
Functional connectivity and graph analysis in DAT-KO, DAT-HET and WT rats. **A** Network-based statistic (NBS) results demonstrate a pattern of strong cortical hyperconnectivity and midbrain hypoconnectivity in DAT-KO compared to WT rats. Significantly stronger connections in DAT-KO rats are illustrated in red (stringent threshold p_pt_ < 0.01, p_NBS_ < 0.05) and orange (lenient threshold p_pt_ < 0.05, p_NBS_ < 0.05), while significantly weaker connections in the DAT-KO group are illustrated in blue (lenient threshold p_pt_ < 0.05, p_NBS_ < 0.05). **B** Normalized local clustering coefficients on the whole-brain network show a pattern of significant increase in frontal regions and a decrease in midbrain dopaminergic regions and parts of the thalamus in DAT-KO compared to WT rats. **C** Small-world propensity decreased across genotypes from WT to DAT-HET and DAT-KO animals. **D** Small-world propensity negatively correlated with total distance moved and was positively associated with relative dorsal striatal volume in DAT-KO rats. **E** Both global and local efficiencies demonstrated a robust decrease in DAT-KO animals. **F** Robustness against targeted and random failures showed a prominent reduction in DAT-KO rats. Glob. Eff. global efficiency, Mean Loc. Eff. mean local efficiency, Rob. (targ.) robustness against targeted attacks; Rob. (rand.) robustness against random attacks; Small-World Prop. small-world propensity; TBV total brain volume. ^#^*p* < 0.10; *p < 0.05; **p < 0.01; ***p < 0.001. All illustrated metrics are corrected for differences in isoflurane levels (for details, see Supplement). For brain regions abbreviations see Fig. 3.

Remarkably, NBS demonstrated functional changes within the same loop that revealed structural changes: the cortico-striatal circuit displayed pronounced hyperconnectivity in DAT-KO compared to WT rats (p_NBS_ < 0.05, p_pt_ < 0.05 (orange connections), p_pt_ < 0.01 (red connections), Fig. 4A). Main affected connections included OFC, motor, cingulate and dorsostriatal regions. In contrast, several thalamic regions and particularly VTA showed hypoconnectivity with cortex and striatum (p_NBS_ < 0.05, p_pt_ < 0.05 (blue connections)) (Fig. 4A). Analysis between other subgroups revealed no significant differences (Fig. S9).

Similar to NBS results, local clustering coefficient, quantifying information exchange within a node’s vicinity, presented a comparable pattern: while frontal regions (OFC, cingulate, M1, M2) exhibited increased clustering in DAT-KO rats, midbrain (VTA, SN) and thalamus showed a significant decrease (Fig. 4B).

### Graph analysis shows whole-brain network alterations in DAT-KO rats

DAT-KO rats demonstrated robust deficiencies in whole-brain function compared to WT, and, to a weaker extent, to DAT-HET rats. Most importantly, SWP, characterizing the optimal small-world organization present in healthy brain, was remarkably reduced in DAT-KO group (F_2,32_ = 11.55, *p* < 0.001) (Fig. 4C). Correlating SWP with volumetric and behavioral measures, we detected an association between impaired SWP and striatal volume loss in DAT-KO rats (*r* = 0.62, *p* < 0.05), and a negative correlation of SWP with hyperactivity (*r* = −0.59, *p* < 0.05) (Fig. 4D).

Global efficiency, representing the ability to combine specialized information from distributed regions, was reduced in DAT-KO rats (F_2,32_ = 15.03, *p* < 0.001); mean local efficiency, measuring local information exchange, was also decreased, albeit less strongly (F_2,32_ = 4.79, *p* < 0.05) (Fig. 4E). Further, network’s robustness to withstand targeted or random failures was negatively affected (F_2,32_ = 3.57, *p* < 0.05; F_2,32_ = 8.59, *p* < 0.001) (Fig. 4F).

Correlation of SWP with local clustering coefficient and degree demonstrated that the impairment of whole-brain function in DAT-KO rats was largely driven by frontal-midbrain decoupling (Fig. S10).

### Spectroscopy reveals elevated prefrontal myo-inositol levels

**Fig. 5:**
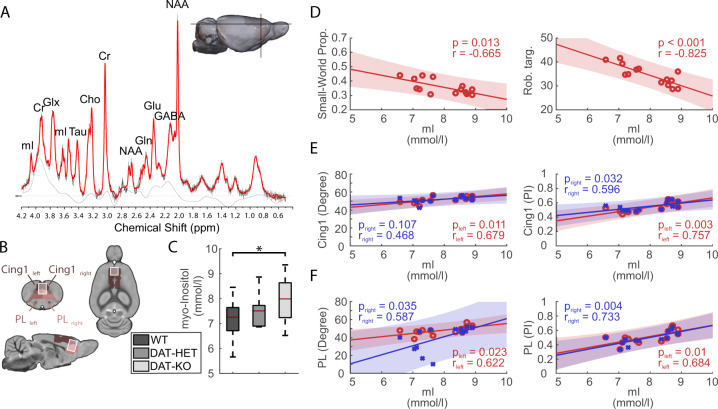
Myo-inositol levels in the prefrontal cortex of DAT-KO rats and correlation of myo-inositol to graph metrics. **A** A typical point-resolved spectroscopy spectrum acquired from the prefrontal cortex of one of the rats in the current study and overlaid by the LC Model fit curve. Peak resonances are shown for N-acetylaspartate (NAA), total creatine (Cr), total choline (Cho), myo-inositol (mI), glutamate (Glu), glutamine (Gln), combined glutamate and glutamine (Glx), gamma-aminobutyric acid (GABA) and tau (Tau). **B** The voxel localization in our study covered predominantly cingulate and prelimbic areas in the left and right hemispheres. **C** Myo-inositol exhibited significantly higher levels in DAT-KO compared to WT rats. **D** Partial correlation (with isoflurane as covariate) of myo-inositol with global graph metrics (small-world propensity and robustness against targeted attacks). **E–F** Partial correlation (with isoflurane as a covariate) of myo-inositol with degree and participation index (PI) of cingulate and prelimbic areas. For brain regions abbreviations see Fig. 3, for other abbreviations see Fig. 4. ppm parts per million. *p < 0.05.

Considering the relevance of the prefrontal cortex for multiple psychiatric disorders, we focused our spectroscopy analysis on prelimbic-cingulate cortex (Fig. 5A, B). MR-spectroscopy revealed significantly increased myo-inositol levels in DAT-KO compared to WT rats (F_2,32_ = 3.52, *p* < 0.05) (Fig. 5C). Higher levels of myo-inositol correlated with impaired SWP (*r* = −0.665, *p* = 0.013) and robustness (*r* = −0.825, *p* < 0.001) in the DAT-KO rats (Fig. 5D) suggesting a relationship with whole-brain network dysfunction. Further, elevated myo-inositol levels were associated with increased degree and participation index of prelimbic-cingulate cortex (Fig. 5E-F).

## Discussion

Our multimodal imaging investigation of DAT-KO effects identified robust spatially distributed structural and functional brain alterations encompassing motor, limbic and associative loops that were highly consistent across imaging modalities and behaviorally relevant. Most prominently, we detected a volume loss in dorsal striatum as a central hub of all loops, co-occurring with a distinct volume gain in other key regions including prefrontal and motor cortices, thalamus and cerebellum. Specific negative correlation between striatal and cerebellar volumes points towards a potential neurodevelopmental compensation. Volume alterations of both striatum and cerebellum were associated with hyperactivity and repetitive behavior in DAT-KO animals, and for striatum additionally with SWP as a feature of efficient brain organization, thus suggesting these dysfunctions as core neural mechanisms underlying behavioral deviances. FC and graph analyses revealing opposite patterns in frontal and midbrain regions suggest a decoupling mechanism, potentially explaining action inhibition control and persistent non-goal-directed behavior in DAT-KO animals. Elevated myo-inositol in the prelimbic-cingulate cortex and its association with reduced SWP and robustness indicate an additional pathway of how DAT silencing impairs whole-brain function. Considering the transdiagnostic behavioral deviations in this model, our study helps to unravel the neural mechanisms of developmental hyperdopaminergia underlying many neuropsychiatric diseases.

### Behavioral and structural alterations suggest impairment of motor and associative-limbic circuits

Our behavioral investigation of DAT-KO rats revealed two prominent phenotypes: (a) dominant hyperactivity, (b) repetitive non-goal directed behavior, expressed as circular movements and quantified as center avoidance. Both features correlated with striatal volume loss and cerebellar volume gain in the DAT-KO rats, suggesting them as key alterations underlying these patterns.

Detection of hyperactivity in DAT-KO rats replicates previous investigations [15] and presumably results from increased dopamine levels in dorsal striatum mediating complex motor functions [40]. Prolonged and excessive dopamine exposure is neurotoxic leading to altered synaptic scaffolding and postsynaptic neurodegeneration [41]. Our finding of striatal volume loss is consistent with data in DAT-KO mice which showed that decreased striatal volume was largely explained by loss of GABAergic interneurons [42]. The degeneration of interneurons might contribute to hyperactivity and observed cortical hyperconnectivity via disinhibition. Also, striatal volume reduction could be caused by decreased spine density, as shown in DAT-KO mice [43], possibly resulting from reduced BDNF expression [15].

Our whole-brain analytical approach expands the explanation of hyperlocomotion as mediated by striatum to a circuit level, since additional parts of the motor system displayed prominent changes. More specifically, we detected a robust volume increase in the cerebellar lobules I-IV, key regions of motor processing [44], and in M1/M2 of DAT-KO rats. Motor cortex and cerebellum do not contain dopamine receptors in such high density as striatum [45], thus possibly avoiding the neurotoxic effects of increased dopamine flux. Both anticorrelation between cerebellar and dorsal striatal volumes and positive association between cerebellar volume and locomotion point towards a potential neurodevelopmental compensation for striatal loss by greater engagement of the cerebellar part of the loop. This compensation possibly reflects a strategy to minimize the adverse influence of DAT-KO on motor function. It appears to have spared motor cortex, as its volume did not correlate with either value. While motor cortex directly causes motor output [46], striatum and cerebellum gate and coordinate movement initiation [40, 47]. Therefore, developmental neurocompensation through recruitment of the cerebellum rather than the motor cortex, presumably occurring during adolescence, would be functionally justified. Evidence from imaging studies demonstrating cerebellar compensation in other dopaminergic disorders [48] supports this notion.

Repetitive non-goal directed behavior, expressed as center avoidance, represents not only a motor feature, but rather a more complex compulsive-like phenomenon largely governed by alterations in the associative-limbic loop [49]. Traditionally, center avoidance was considered in the context of anxiety, however behavioral data in DAT-KO animals in this regard are unclear. While higher corticosterone levels and increased stress susceptibility in female DAT-KO rats [50] support this notion, reduced freezing in males exposed to fear-associated context [18] questions this idea. It appears more probable that center avoidance adds to a series of compulsive-like features in DAT-KO rats [15, 17, 18], indicating emotional dysregulation driven by disturbances in the associative-limbic loop.

The volume of striatum, as a part of the associative-limbic loop, correlated positively with center time, suggesting that its reduction might be implicated in center avoidance, whereas the opposite was true for cerebellum. Remarkably, cerebellar correlation with center avoidance was even stronger than with hyperlocomotion. Contrary to the dominant view that cerebellum is mostly concerned with motor coordination, studies show that its lateral regions are occupied by maps to associative areas and mediate fundamental cognitive and emotional functions, including attentional control and action planning [51]. Cerebellum has remained unexplored in animal models of ADHD, emphasizing the importance of our findings.

Our functional imaging data support two hypotheses on neural mechanisms for compulsive traits in DAT-KO. First, compulsiveness may result from deficits in inhibitory action control, related to dopaminergic imbalance within the frontal-midbrain circuit [52] (discussed in the next section *Frontal-midbrain decoupling and impairment of whole-brain function*). Secondly, a recent study explains compulsiveness by enhanced OFC-dorsostriatal connectivity [53]. In our study OFC, as well as cingulate and thalamic areas, demonstrated increased connectivity to dorsal striatum and motor areas, along with structural volume gain. OFC is proposed to potentiate synaptic activity in the dorsal striatum, the site of habit formations [53], and is additionally interconnected with cingulate cortex and mediodorsal thalamus. Cooperatively they support goal-directed behavior and flexibility by updating old strategies during dopamine-mediated learning [54, 55]. Cognitive rigidity is a hallmark of many neuropsychiatric disorders including OCD [56] and schizophrenia [57] and is pronounced in DAT-KO rats, as demonstrated by inflexibility in dynamic reward tasks [17]. Considering the role of each region in adapting to dynamically changing environments, our data support the notion that morphological and functional alterations within the cortico-striatal-thalamic circuitry contribute to cognitive rigidity and persistent behavior in DAT-KO rats.

### Frontal-midbrain decoupling and impairment of whole-brain function

We corroborated our findings of reciprocal volume changes in prefrontal and subcortical dopaminergic regions with functional analyses demonstrating cortical hyperconnectivity along with midbrain hypoconnectivity. This feature was paralleled by alterations in graph-theoretical markers of local information exchange, being increased in cortical and decreased in dopaminergic midbrain and in thalamic regions. In sum, these data indicate a decoupling between frontal and midbrain parts of motor and associative-limbic loops in DAT-KO rats. Proper dopamine function is essential for information transfer by coding salience and reward prediction error [58], and for synaptic plasticity [59]. DAT dysfunction interferes with this process, thereby providing a potential background for midbrain-cortical decoupling. Intact frontal-midbrain communication and dopamine balance are necessary prerequisites for action inhibition control [60, 61]. Behavioral disinhibition represents an overlapping feature of impulsivity (in ADHD) and compulsiveness (in OCD), presumably resulting from dopaminergic imbalance and curtailed top-down control [58]. Also, disruptions in prefrontal connectivity have been repeatedly linked to altered dopamine function in schizophrenia [62, 63]. Importantly, a manganese-enhanced MRI study on DAT-KO mice points to the same direction by demonstrating a disrupted prefrontal-mesolimbic transport [64].

The prefrontal disturbance is corroborated by spectroscopy data showing increased myo-inositol in prelimbic-cingulate cortex of DAT-KO rats. Myo-inositol, an important structural component of cell membranes, plays an important role in the neuronal phosphatidylinositol second messenger system [65]. Positive association with increased degree and participation index of prefrontal regions indicates its contribution to the prefrontal hyperconnectivity observed in DAT-KO rats. Supporting our notion of DAT-KO as a transdiagnostic model, frontal changes in myo-inositol emerge in mania [66], OCD [67], and schizophrenia [68]. Moreover, elevated myo-inositol is a neuroinflammation marker and has been detected in the anterior cingulate cortex of chronic dopaminergic stimulants users [69], possibly representing a neurotoxic condition under prolonged dopamine exposure.

On the whole-brain level, DAT-KO rats exhibited ample reductions in several graph metrics, revealing less efficient whole-brain organization, expressed by reduced SWP, decreased global and local information processing, and reduced robustness against network failures. These alterations suggest compromised information transmission and stability, potentially resulting in cognitive deficits, such as working memory impairment and inflexibility. Impaired SWP was associated with hyperlocomotion, elevated myo-inositol, striatal volume loss and frontal-midbrain decoupling, further emphasizing fronto-striatal-midbrain alterations as a central pathomechanism for whole-brain dysfunction.

Finally, comparing our findings to other genetic variations in chromosomal regions associated with schizophrenia and ADHD [31, 70], we found particular resemblances with 1q21.1 microdeletion including microcephaly, enlarged cerebellar and reduced hippocampal volume [31]. Mice carrying 1q21.1 deletion also exhibit altered mesolimbic dopaminergic transmission and hypersensitivity to amphetamine [71], similarly to DAT-KO rats [15]. It is possible that convergence of brain structural findings across these genetic models indicate common neural mechanisms behind ADHD and schizophrenia features.

### Limitations

Direct translatability of our main results to clinical diseases is limited by the inherent characteristic of the constitutional DAT-KO model, demonstrating a sustained region-unspecific hyperdopaminergia, which is unlikely to occur in any of the disorders to such an extent. Future work should address effects of conditional knockouts, distinct developmental stages, and gene-environment interactions. For this, our findings might serve as a starting point towards better understanding the complex role of hyperdopaminergia in neuropsychiatric diseases.

The results of our study are limited to male rats. However, based on recent evidence of comparable behavioral phenotypes in female DAT-KO rats [50, 72] and of higher DAT activity and striatal dopamine release in female rats in general [73], we would expect analogous or conceivably even more severe effects induced by greater dopaminergic excitotoxicity.

Unequal sample sizes (*N* = 14 DAT-KO, *N* = 8 DAT-HET, *N* = 14 WT) limit the power to detect specific differences in DAT-HET rats. However, complying with the main aim of our study to unravel the pathomechanistic pathways behind the phenotypes of DAT-KO rats, more detailed investigations of the associations between brain imaging endophenotypes and behavioral features in this group were indispensable. This required larger sample sizes to reach an adequate statistical power.

## Conclusion

Our multimodal imaging genetics approach of DAT-KO rats revealed prefrontal-midbrain decoupling and striatal-cerebellar compensation as two key features of developmental DAT blockade. Both findings demonstrated strong behavioral relevance and were highly consistent across imaging modalities. Thus, our data mechanistically connect developmental hyperdopaminergia to systems-level brain changes, underlying impaired action inhibition control and resulting in neuropsychiatric symptoms relevant for ADHD, schizophrenia and OCD.

## Supplementary information

Supplemental Material

## Data Availability

Codes used for graph analysis and NBS are freely available from the Brain Connectivity Toolbox [37]. In-house MATLAB codes for DBM, statistical analysis and illustration of results are available upon request.
