# Supplementary material for: Dopamine transporter silencing in the rat: systems-level alterations in striato-cerebellar and prefrontal-midbrain circuits
Source: Mol Psychiatry. 2022 Mar 4;27(4):2329–39. doi: 10.1038/s41380-022-01471-4 (PMC9126810; doi:10.1038/s41380-022-01471-4)
Supplement: Supplementary file 1 — Supplemental Material [file 41380_2022_1471_MOESM1_ESM.docx]

**SUPPLEMENT**

**Animals**

Three groups consisting of N=14 DAT-KO (average weight 230 g during imaging), N=8 DAT-HET (average weight 319 g), and N=14 wild type (WT) (average weight 316 g) male rats participated in the experiments. Group sizes (N=14 per group) were chosen based on prior knowledge from our previous studies [1-3] and are toward the high end of the range typically used in animal MRI experiments. The experimenters were blinded throughout the experiment, although the difference in body weight of one group (DAT-KO) indicated the genotype.

The animals were randomly assigned to their home cages, where they were housed in groups of 3-4 animals per cage in standard type IV cages with aspen bedding (Ssniff GmbH, Soest, Germany), Sizzle nesting material (Zoonlab GmbH, Castrop-Rauxel, Germany), an aspen wood brick for gnawing (Ssniff GmbH) and a cardboard tunnel (Ssniff GmbH) at a temperature of 22-24°C and 12-h light–dark cycle (lights on at 07:00 a.m.). Food (Altromin International, Lage, Germany) and tap water were available *ad libitum*. Rats were moved from the quarantine room into their housing room 5 weeks after arrival. At the end of the experiments the rats were killed. No animals were excluded from the study.

**Behavioral testing**

**Open field test.** After an acclimatization to the experimental room for 15 min, an individual rat was placed for 30 min into an unfamiliar arena (50 x 50 cm) illuminated from above (15 Lux). The apparatus was thoroughly cleaned with 70% ethanol between sessions. Locomotor activity was recorded and ‘movement’, ‘total distance moved’, ‘velocity’, ‘center time’, ‘distance to walls’ were analyzed using EthoVision® XT (Noldus, Wageningen, Netherlands).

**Prepulse inhibition test.** PPI was assessed in startle chambers (SR-LAB, San Diego Instruments, San Diego, USA), as described previously [4]. The testing chamber was thoroughly cleaned with 70% ethanol between trials. Before the first exposure to acoustic startle or prepulses, the animals were acclimatized to the apparatus with white background noise only (65 dB sound pressure level) for 5 min. Startle responses were evoked by 115 dB white noise of 40 ms duration. Prepulses were presented 100 ms before the startle stimulation with four different intensities (72, 76, 80, 84 dB, duration 20 ms). Initially, five startle stimuli were presented (excluded from analysis), and subsequently the test program pseudorandomly displayed six different trial types (startle stimulus, prepulse-startle combinations or background noise alone). Each trial type was presented 10 times. The inter-trial interval varied between 10 and 20 s. PPI was calculated as the individual percentage decrease of the startle response magnitude in trials with preceding prepulse:

100 * (1 – (mean startle amplitude on prepulse/mean startle impulse on pulse alone trials)).

Due to technical problems of the testing startle chamber, prepulse inhibition data were available in the following numbers for WT/DAT-HET/DAT-KO: 72 dB (13/7/7), 76 dB (13/7/10), 80 and 84 dB (13/7/8).

**MRI acquisition**

The MRI experiments were carried out at a small-animal 9.4 Tesla MRI scanner (Bruker BioSpec, Ettlingen, Germany) with Avance III hardware, BGA12S gradient system (maximum strength 705 mT/m) and Paravision 6 software. The cryocoil consists of an anatomically shaped four-channel receive-only low temperature array combined with quadrature whole-body volume transmitter operating at room temperature. The rats were initially anesthetized with 4% isoflurane (Baxter Deutschland GmbH, Unterschleissheim, Germany) in a mixture of 70% N2 and 30% O2. After positioning in the scanner, isoflurane level was reduced to 2.5%, and medetomidine (Domitor, Janssen-Cilag, Neuss) was injected as a bolus (0.03 mg/kg, s.c.). The isoflurane level was reduced to 0.5% during acquisition of the structural image and the animals received continuous medetomidine (0.06 mg/kg/h). We monitored sedation depth throughout the experiment via recording the respiratory and cardiac parameters at 10-ms resolution using a signal breakout module (Small Animal Instruments Inc., NY, USA) and a 4-channel recorder (Velleman® N.V., Gavere, Belgium). Body temperature was maintained at 37°C throughout the session.

Spectroscopy data were acquired using a PRESS (point resolved spectroscopy) sequence (repetition time/short echo time (TR/TE) 4000/10 ms, 256 averages, total acquisition time 17 min per spectrum) from a 12 µl volume (3 x 2 x 2 mm³) placed in the prefrontal (prelimbic-cingulate) cortex and angulated to exclude any partial volume. Water suppression was done with variable pulse powers and optimized relaxation delays (VAPOR). Voxel shimming was achieved by FieldMap-based optimization to ensure a water line full-width-at-half-maximum < 13Hz.

Structural scans were performed using T2-weighted rapid acquisition with refocused echoes (RARE) sequence (RARE factor 16, TR/TE 1200/50 ms, flip angle 90°, voxel dimensions 0.15 x 0.15 x 0.288 mm^3^, acquisition time 22 min). The rsfMRI time series were acquired using a T2*-weighted echo-planar imaging (EPI) free induction decay sequence (TR/TE 1500/17.5 ms, flip angle 60°, field of view 35 x 35 mm^2^, voxel dimension 0.365 mm in plane, 32 coronal slices, slice thickness 0.5 mm with 0.2 mm interslice gap, 350 acquisitions over 8.75 min, 8 dummy scans). A 3D double gradient echo FieldMap sequence (TR/short-TE/long-TE 20/1.7/5.7 ms) was acquired for correction of geometric distortions before each rsfMRI.

**Deformation-based morphometry**

The acquired structural images were resized by a factor of 10 (only the matrix defining the image position in space was changed for better SPM compatibility) and registered to SIGMA template [5] in the common standard space of the atlas (rigid body registration without data reslicing). Coregistered images were skull-stripped and segmented, separating gray matter, white matter and cerebrospinal fluid using a Gaussian mixed model and tissue probability masks from the SIGMA atlas [5]. The tissue segmentation allows to create a group-specific DARTEL template and normalize all data into this template space, thereby obtaining individual deformation maps with Jacobian values.

For the regional analyses based on selected atlas regions, the individual normalized Jacobian maps were overlaid with a digital rat atlas [5] and the values for selected regions were extracted, normalized to the individual total brain volume and used for further analyses (e.g. one-way ANOVA with *post hoc* Tukey-Kramer test or correlation analyses).

Brain extraction was performed using an in-house tool with an adapted algorithm based on a 3D pulse-coupled neural network [6], incorporating prior data optimization including contrast and edge enhancement and intensity normalization. Afterwards, we additionally checked every brain for the quality of extraction.

**Graph theoretical analysis**

Mean regional BOLD time courses were used to calculate Pearson’s correlation matrices which were normalized by their maximum weights and used to compute functional connectivity graphs. Topological characteristics of these graphs were calculated using Brain Connectivity Toolbox (version 2016-01-16) [7]. We focused on a 5–40% range (1% step) of network densities for calculating metrics using binarized networks. We ensured that networks maintained connectedness, meaning the ability of every node to reach every other node. Normalization was done using comparable random graphs preserving number of nodes, degree distribution, and connectedness as null models [8]. Mean metric values across thresholds were used to identify systematic effects that are not dependent on a specific threshold. Modular partitions of the individual networks were detected using the Newman algorithm [9]. Degree (number of connections for a given node), local clustering coefficient (number of connections between nodal neighbors, normalized to the maximum number of possible connections) and participation index (ratio of intermodule strength to the total nodal strength) were assessed to explore a specific region’s role in the network structure. To explore changes in global topology, we calculated small world propensity (deviation of a network’s clustering coefficient and characteristic path length from both lattice and random networks constructed with the same number of nodes and the same degree distribution), global efficiency (average inverse shortest path length), mean local efficiency (mean inverse shortest path length computed on node neighborhood and averaged for all nodes) and robustness against targeted and random attacks (network global efficiency calculated after removal of random or specific nodes (specificity is defined by degree of the nodes)) [10, 11].

**Statistics**

Kolmogoroff-Smirnoff tests were performed to investigate non-normal distribution in all groups (DAT-KO, DAT-HET and WT). No significant deviations could be detected in behavior, regional volume (deformation-based morphometry), global graph metrics, local graph metrics and spectroscopy (p<0.05, FDR-corrected).

O’Brien tests were performed to assess for homogeneity of variances across the groups as a necessary assumption for one-way ANOVA. Importantly, several behavioral metrics (center time, velocity and total distance moved) demonstrated unequal variances between the groups (p<0.05, FDR-corrected), while this could not be found for any of the other metrics. Therefore, non-parametric one-way ANOVA using permutation tests (Matlab function *randanova1* (David Stern (2021) <https://www.mathworks.com/matlabcentral/fileexchange/44307-randanova1>, MATLAB Central File Exchange, retrieved October 15, 2021)) was applied for comparison of the behavioral metrics.

**Results**

There were no significant differences between WT and DAT-HET phenotypes for any parameter in behavior and DBM (**Fig. 2C**).


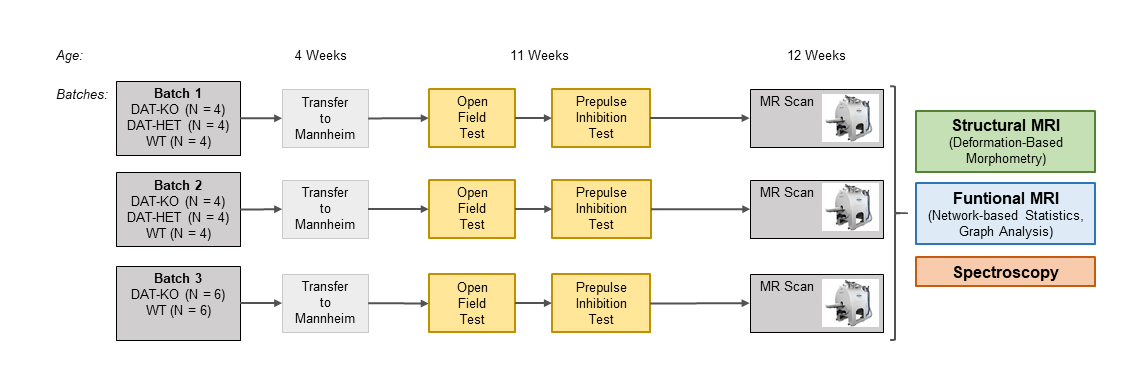


Figure S1. **Experimental design**. Three batches of animals were transferred to Mannheim at the age of 4 weeks. Behavioral tests were performed at the age of 11 weeks, while MR scans followed at the age of 12 weeks.

DAT-KO, dopamine transporter homozygous knockout; DAT-HET, dopamine transporter heterozygous knockout; MRI, magnetic resonance imaging; WT, wild type.


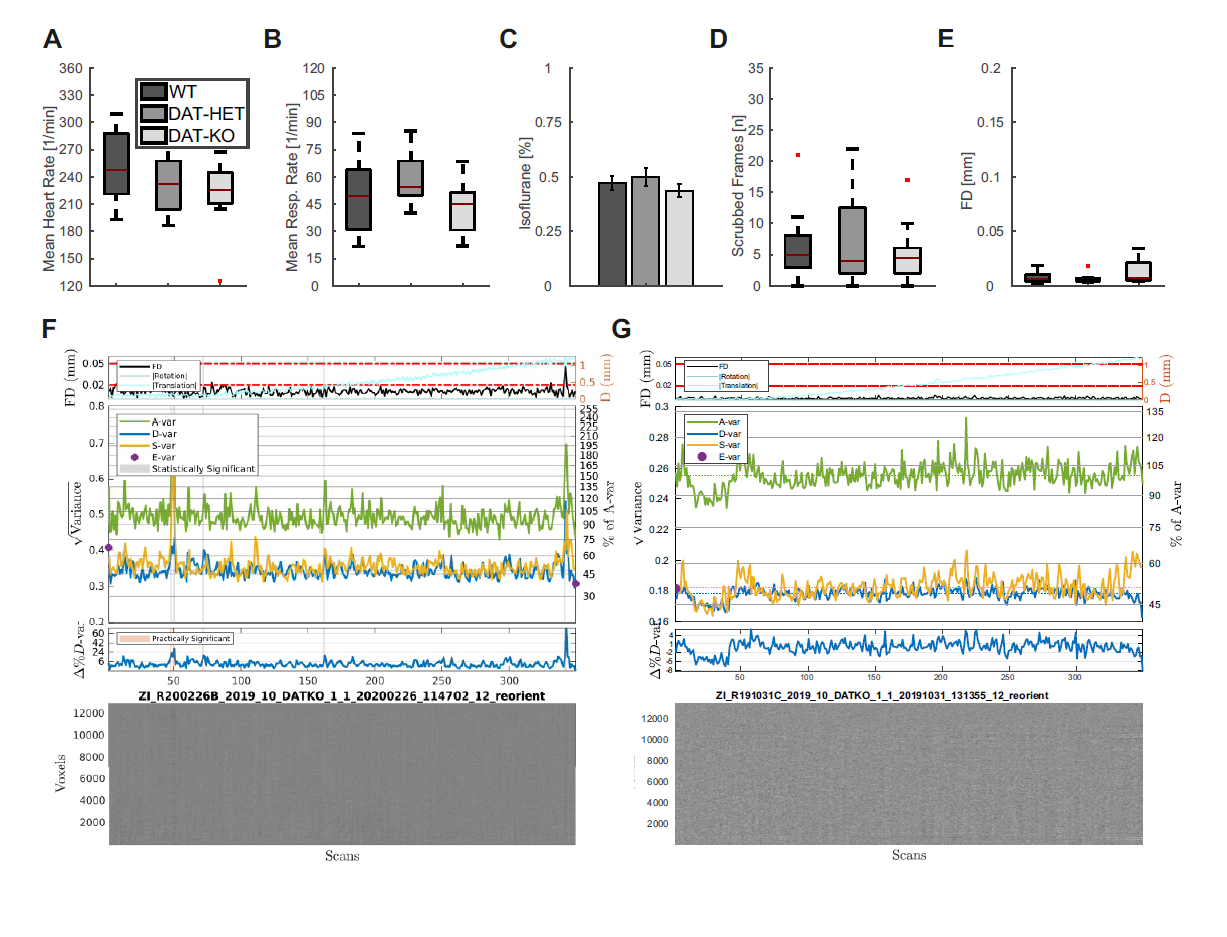


Figure S2. **Physiological parameters, isoflurane dose, motion artifacts (number of scrubbed frames) and framewise displacement for DAT-KO, DAT-HET and WT rats and motion assessment illustrated for two exemplary rats. (A)** Mean values (+/- SEM) of heart rate. **(B)** Mean values (+/- SEM) of respiration rate. **(C)** Mean values (+/- SEM) of isoflurane dose. **(D)** Mean values (+/- SEM) of scrubbed frames. **(E)** Mean values (+/- SEM) of framewise displacement (FD). Results from one-way ANOVA with *post hoc* Tukey-Kramer test for multiple comparison correction show no significant difference (p>0.05) for **(A-E)**. **(F-G)** DSE (D-var, S-var and E-var) and DVARS inference for pre-processed data of two typical animals based on [12]. The upper panel shows four plots, framewise displacement (FD), the DSE plot, the percent delta-value of D-Var, and an image of all brain voxels over time. FD plots show 0.02 mm and 0.05 mm, strict and lenient thresholds, respectively. All time-series plots have frames flagged as significant on the DVARS test marked gray if only statistically significant (5% Bonferroni) and orange if also practically significant (Δ%D-var>5%). Note the motion artefact around frame 50 in **(F)**.

DAT-KO, dopamine transporter homozygous knockout; DAT-HET, dopamine transporter heterozygous knockout; DVARS, spatial standard deviation of data after temporal differencing; SEM, standard error of the mean; WT, wild type.

**
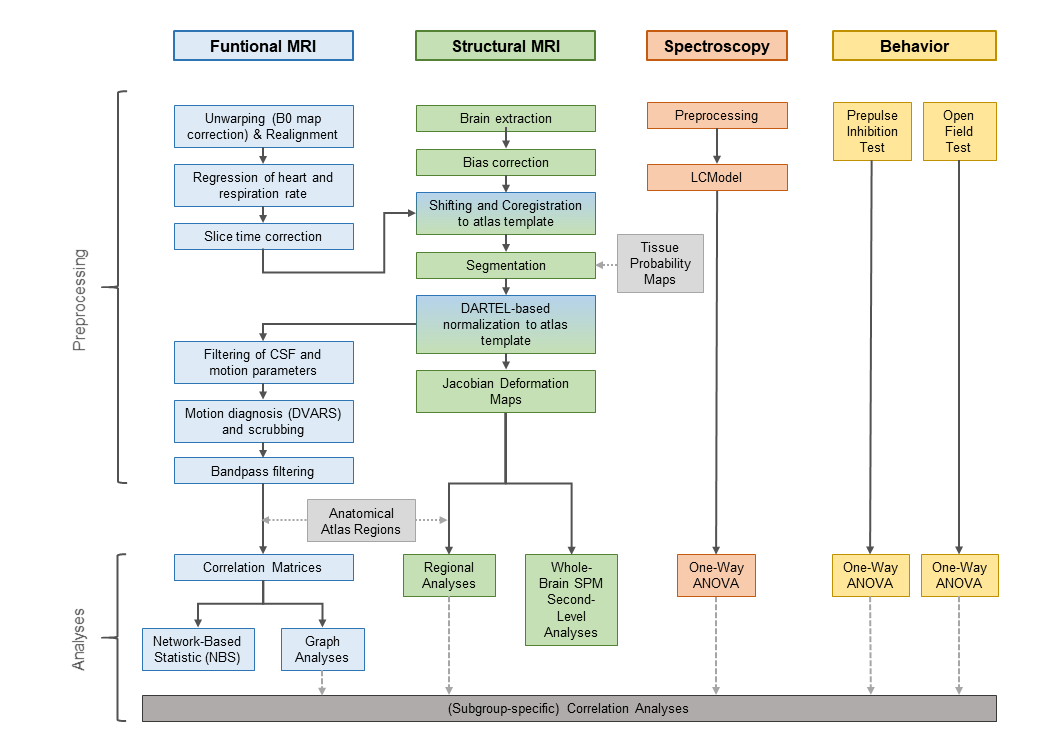
**

Figure S3. **Scheme of preprocessing steps and analyses.**

ANOVA, analysis of variance; CSF, cerebrospinal fluid; DARTEL, Diffeomorphic Anatomical Registration using Exponentiated Lie algebra; DVARS, spatial standard deviation of data after temporal differencing; SPM, statistical parametric mapping (software).


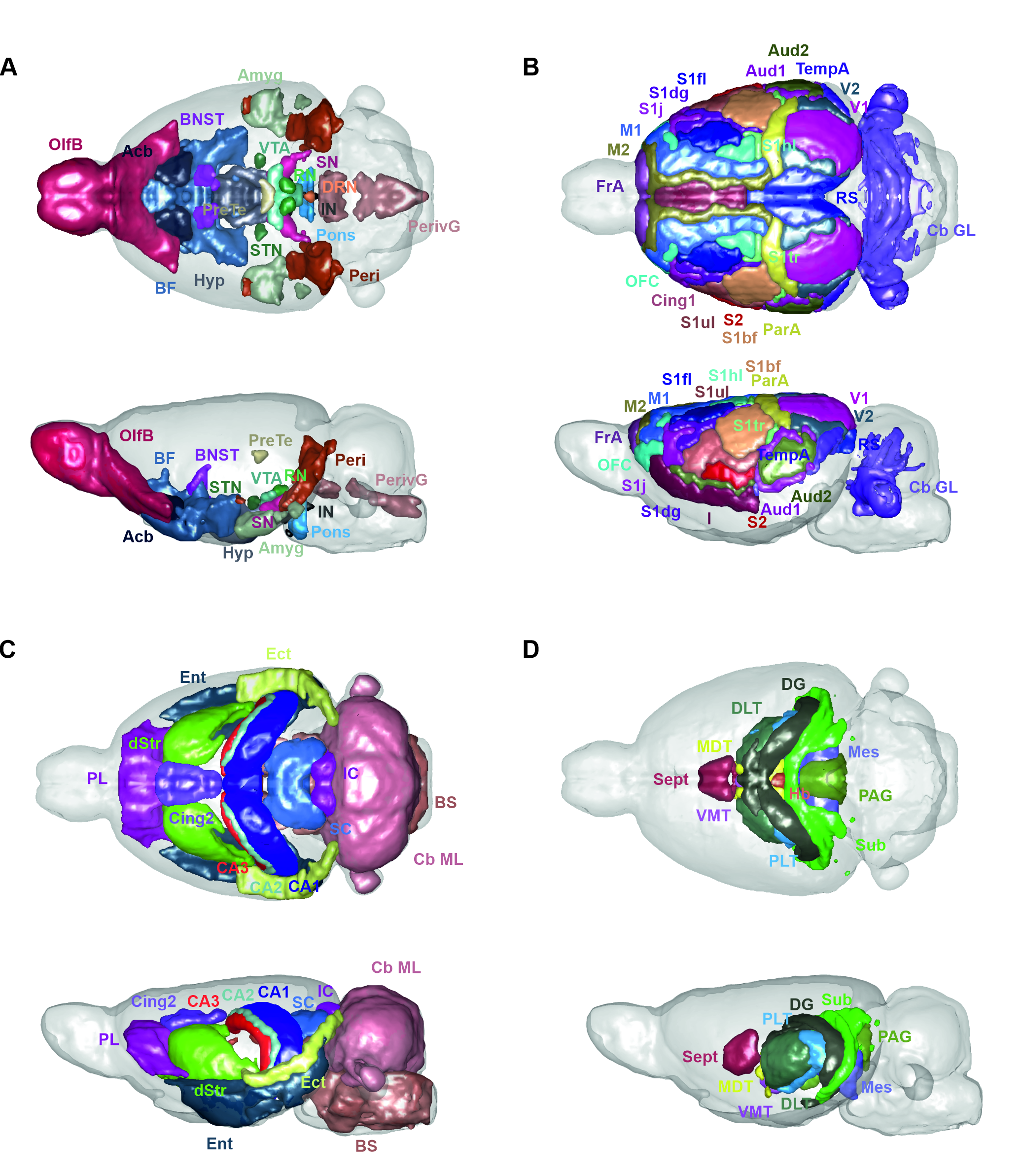


Figure S4. **Atlas parcellation**. Brain parcellation is based on the SIGMA atlas [5].

Acb, nucleus accumbens; Amyg, amygdala; Aud1, primary auditory cortex; Aud2, secondary auditory cortex; BF, basal forebrain; BNST, bed nucleus of the stria terminalis; BS, brainstem; CA1-CA3, fields CA1-CA3 (cornu ammonis) of hippocampus; Cb GL, granular layer of the cerebellum; Cb ML, molecular layer of the cerebellum; Cing1, cingular cortex area 1; Cing2, cingular cortex area 2; DG, dentate gyrus; DLT, dorsolateral thalamus; dStr, dorsal striatum; Ect, ectorhinal cortex; Ent, entorhinal cortex; FrA, frontal association cortex; Hb, habenula; Hyp, hypothalamus; I, insular cortex; IC, inferior colliculus; IN, interpeduncular nucleus; M1, primary motor cortex; M2, secondary motor cortex; MDT, mediodorsal thalamus; Mes, mesencephalon; OFC, orbitofrontal cortex; OlfB, olfactory bulb; PAG, periaqueductal gray; ParA, parietal associative cortex; Peri, perirhinal cortex; PerivG, periventricular grey; PL, prelimbic cortex; PLT, posterolateral thalamus; Pons, pons; PreTe, pretectal region; RN, raphe nucleus; RS, retrosplenial cortex; S1bf, primary somatosensory cortex barrel field; S1dg, primary somatosensory cortex dysgranular region; S1fl, primary somatosensory cortex forelimb region; S1hl, primary somatosensory cortex hindlimb region; S1j, primary somatosensory cortex jaw region; S1tr, primary somatosensory cortex trunk region; S1ul, primary somatosensory cortex upper lip region; S2, secondary somatosensory cortex; SC, superior colliculus; Sept, septal nuclei; SN, substantia nigra; Sub, subiculum; STN, subthalamic nucleus; TempA, temporal associative cortex; V1, primary visual cortex; V2, secondary visual cortex; VMT, ventromedial thalamus; VTA, ventral tegmental area.


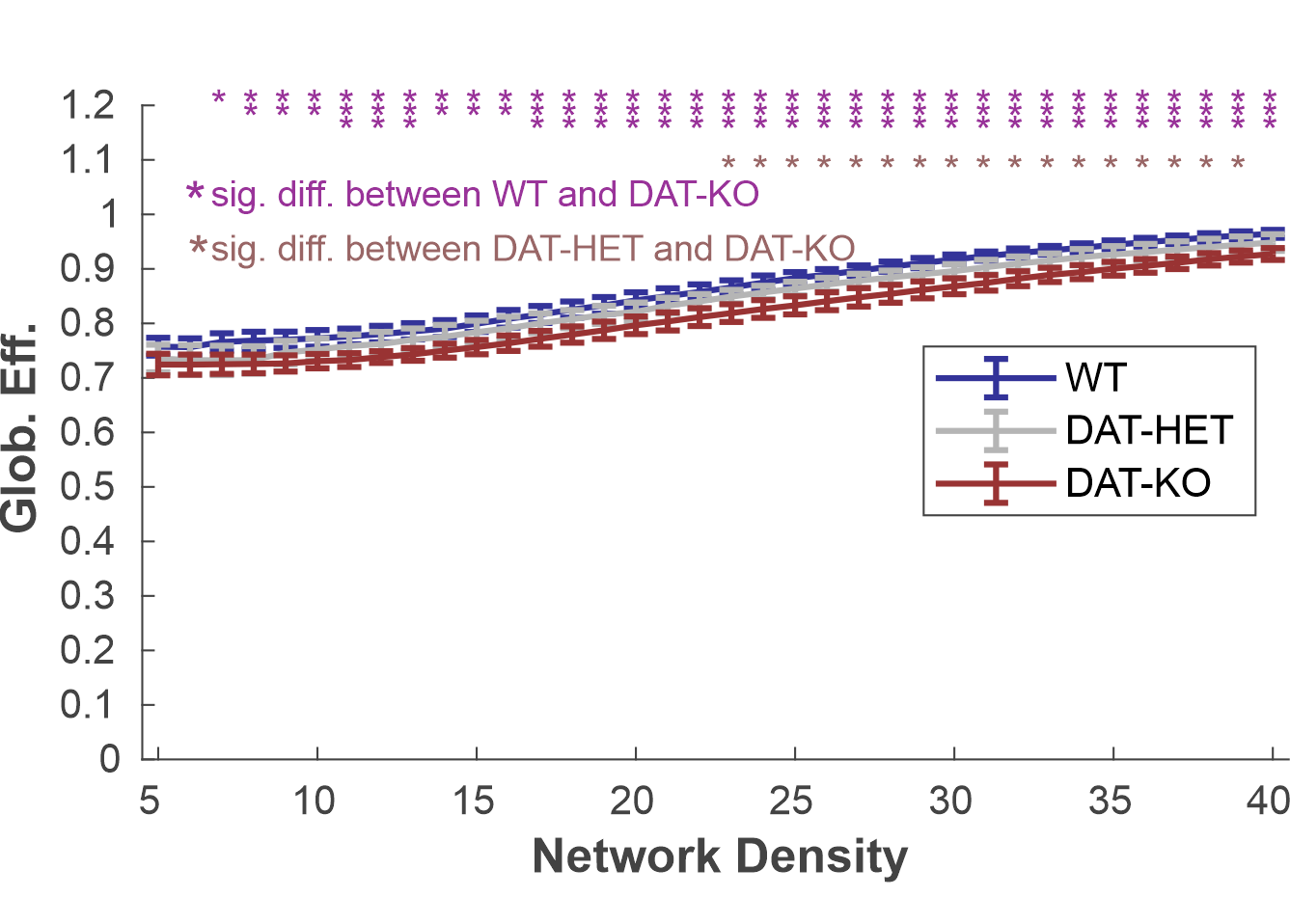


Figure S5. **Stability of group differences for global efficiency over the range of 5-40% thresholds used in the graph theoretical analysis.**

DAT-KO, dopamine transporter homozygous knockout; DAT-HET, dopamine transporter heterozygous knockout; Glob. Eff., global efficiency; WT, wild type.


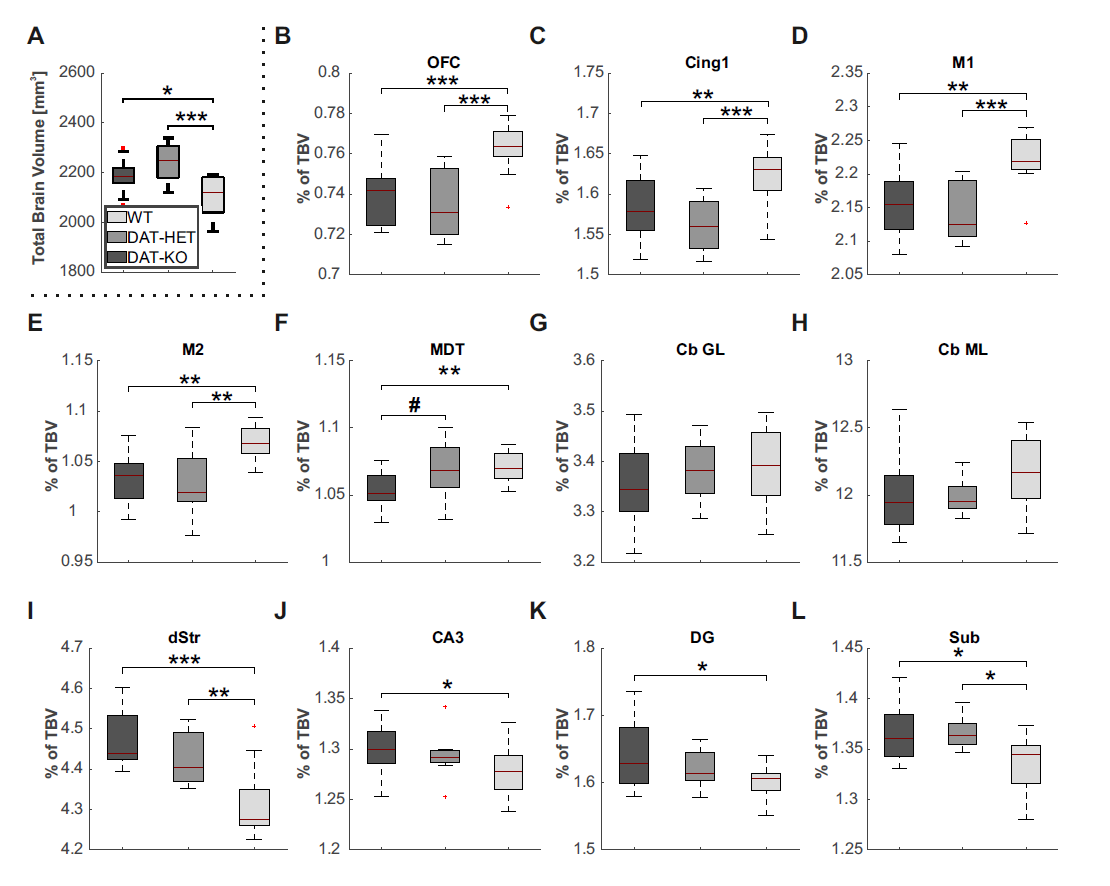


Figure S6. **Group differences in total brain volume and relative regional brain volume. (A)** Mean values (+/- SEM) of total brain volume. **(B-L)** Mean values (+/- SEM) of relative brain volume as percentage of TBV for several brain regions. Results from one-way ANOVA with *post hoc* test (Tukey-Kramer test for multiple comparison correction). # p<0.10; * p<0.05; ** p<0.01; *** p<0.001.

DAT-KO, dopamine transporter homozygous knockout; DAT-HET, dopamine transporter heterozygous knockout; SEM, standard error of the mean; TBV, total brain volume; WT, wild type. Abbreviations for brain regions are the same as in **Figure S4**.


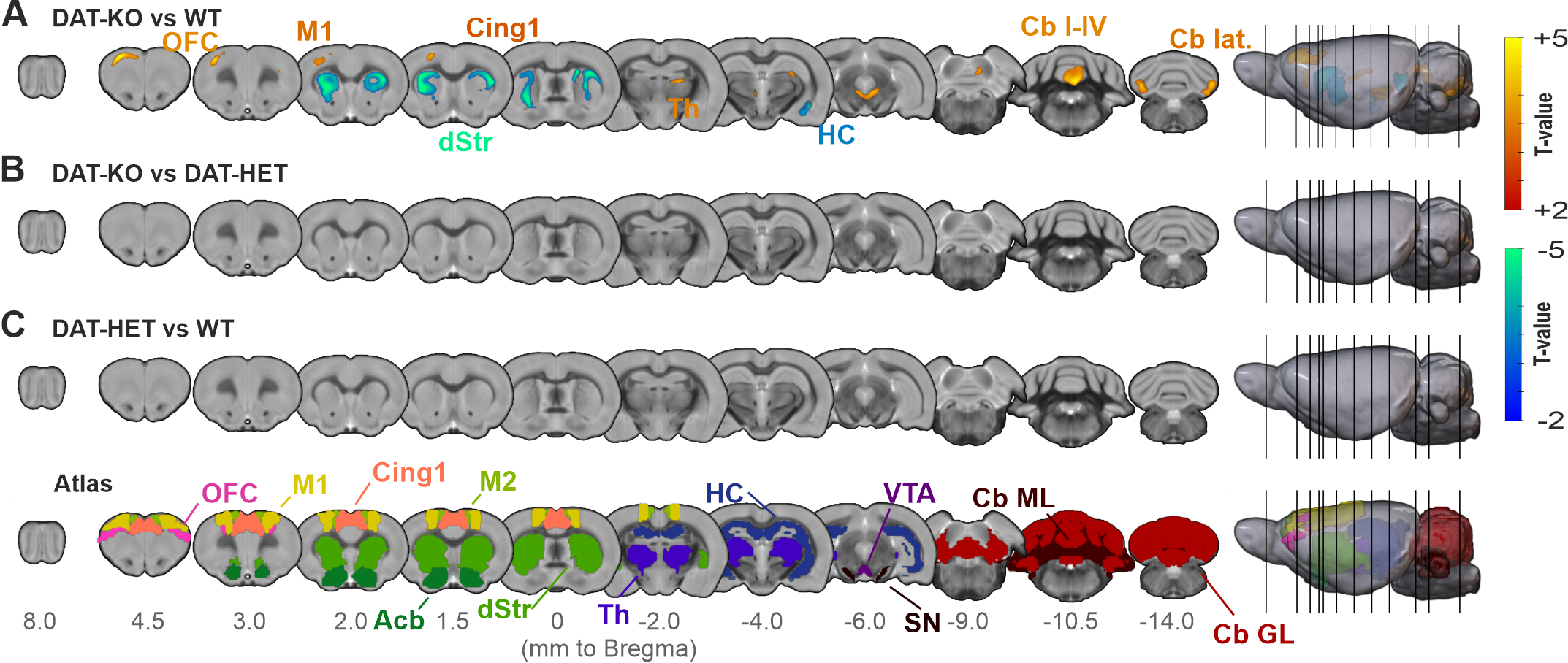


Figure S7. **Brain morphology comparison between DAT-KO, DAT-HET and WT rats and illustration of associative, limbic and motor loops affected by volumetric changes in DAT-KO rats. (A)** Comparison between DAT-KO and WT rats revealed a significant pattern of decreased relative brain volume in DAT-KO rats (blue scale), with clusters covering predominantly striatum, hippocampus and olfactory bulb. In contrast, relative brain volume in orbitofrontal, cingulate and motor regions as well as in thalamus, midbrain and cerebellum were significantly increased (red scale). **(B)**  Comparison between DAT-KO and DAT-HET rats revealed no significant differences between these groups. **(C)** Comparison between DAT-HET and WT rats detected no significant differences between these groups.

All results in **(A-C)** are thresholded at a cluster-defining-threshold level of p<0.001 and only clusters with a size larger than 300 voxels are plotted.

Acb, accumbens; Cb, cerebellum; Cb I-IV, cerebellar lobules I-IV; Cb lat., cerebellum, lateral part; Cb GL, granular layer of the cerebellum; Cb ML, molecular layer of the cerebellum; Cing1, cingulate cortex; dStr, dorsal striatum; HC, hippocampus; M1, primary motor cortex; M2, secondary motor cortex; OFC, orbitofrontal cortex; SN, substantia nigra; Th, thalamus; VTA, ventral tegmental area.

**
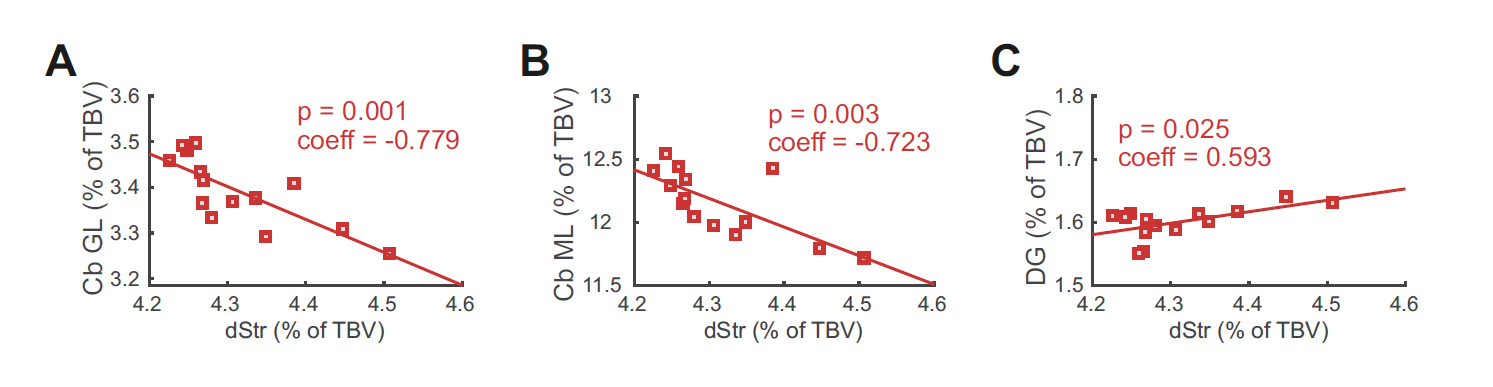
** **
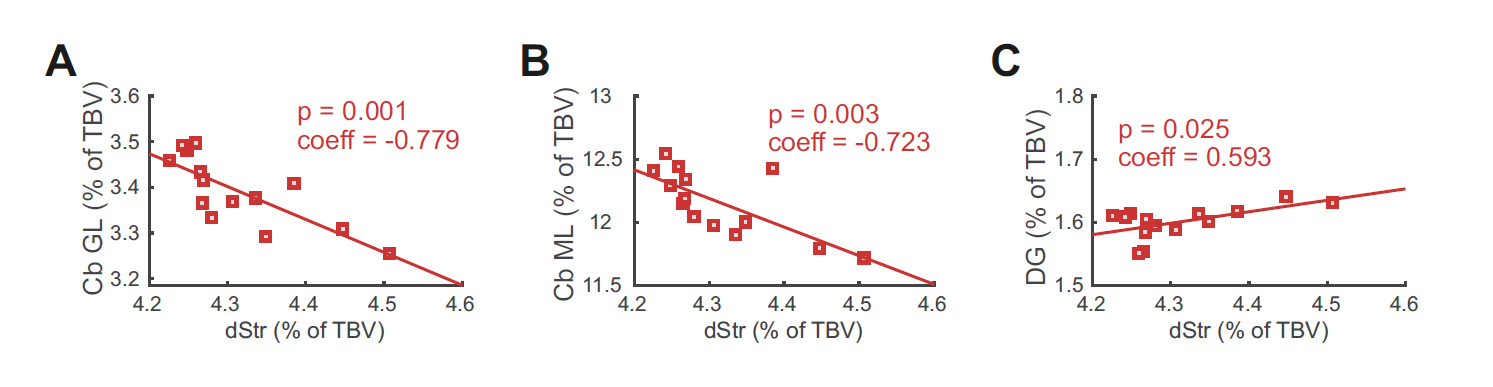
**

Figure S8. **Correlations between dorsal striatal and cerebellar volumes in DAT-KO rats. (A-B)** Group-specific Pearson’s correlation coefficients between regional relative volumes (in percentage of total brain volume) demonstrated a significant anti-correlation between dorsal striatum and cerebellar regions (p<0.001 for granular layer (Cb GL) and p<0.01 for molecular layer (Cb ML)).

coeff, partial correlation coefficient; TBV, total brain volume.


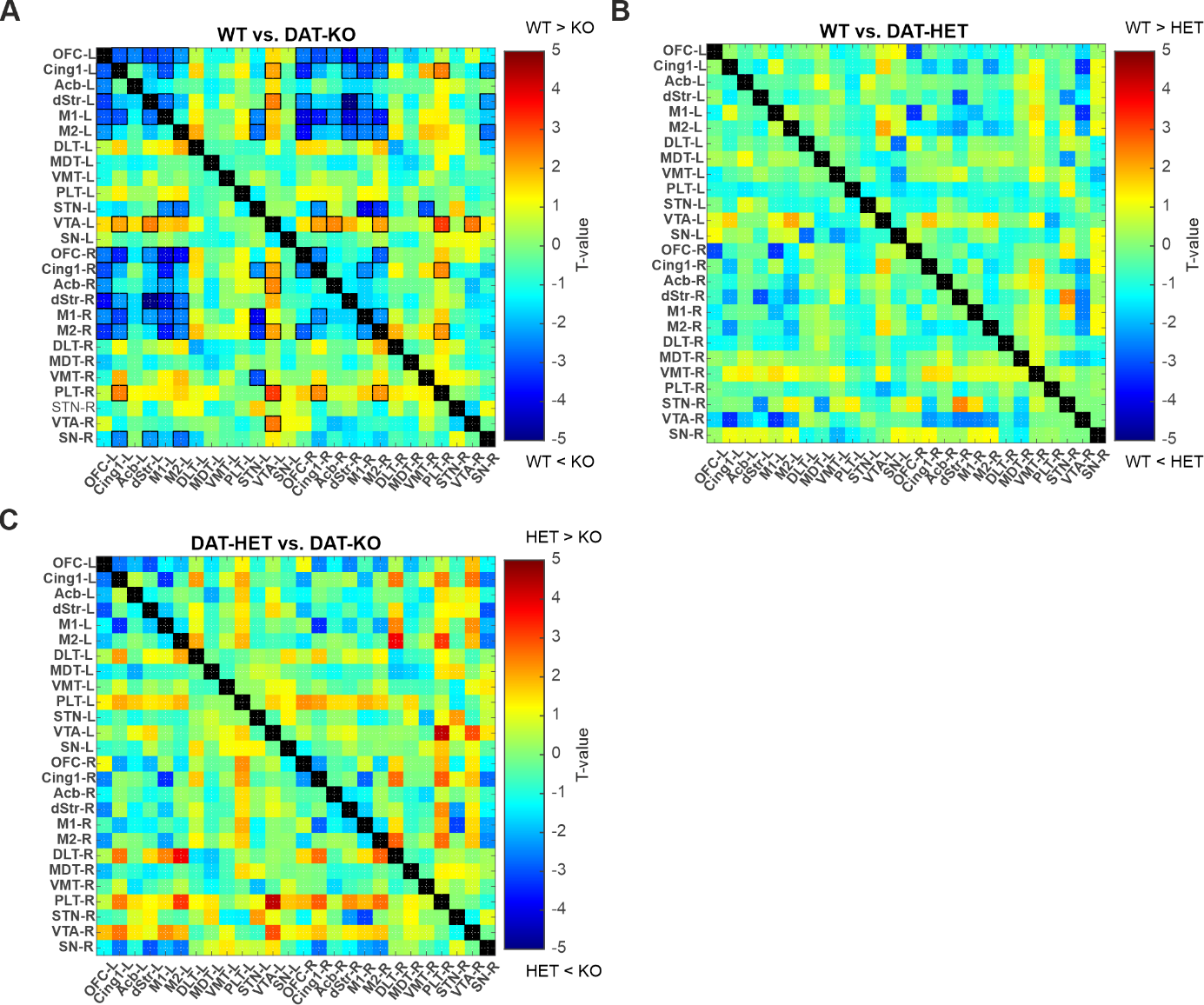


Figure S9. **Network based statistic (NBS) results plotted on matrices of two-tailed T-tests.**

**(A-C)** Results from NBS with a cluster**-**defining threshold of p_pt_<0.05 (corresponding to F_2,26_=4.23 to F_2,20_=4.35) are plotted on T-matrices resulting from group-comparisons between DAT-KO, DAT-HET and WT rats. While NBS defined the cluster by an F-test allowing for different directionalities of the clusters’ connections, the T-matrices illustrate this directionality. Only the comparison between WT and DAT-KO rats **(A)** yielded a significant cluster (p_NBS_<0.05) encompassing various connections marked with black boxes. While VTA demonstrated predominantly lower connectivity in DAT-KO rats (orange to red, WT > KO), prefrontal cortical, dorsal striatal and motor regions showed higher functional connectivity in DAT-KO rats (blue, KO > WT). **(B)** The comparison between WT and DAT-HET rats did not produce significant results. Although a comparable pattern could be observed in the comparison between DAT-KO and DAT-HET **(C)**, it did not reach significance (p_NBS_>0.05).

L-R, left-right hemispheres. Abbreviations for brain regions are the same as in **Figure S4**.


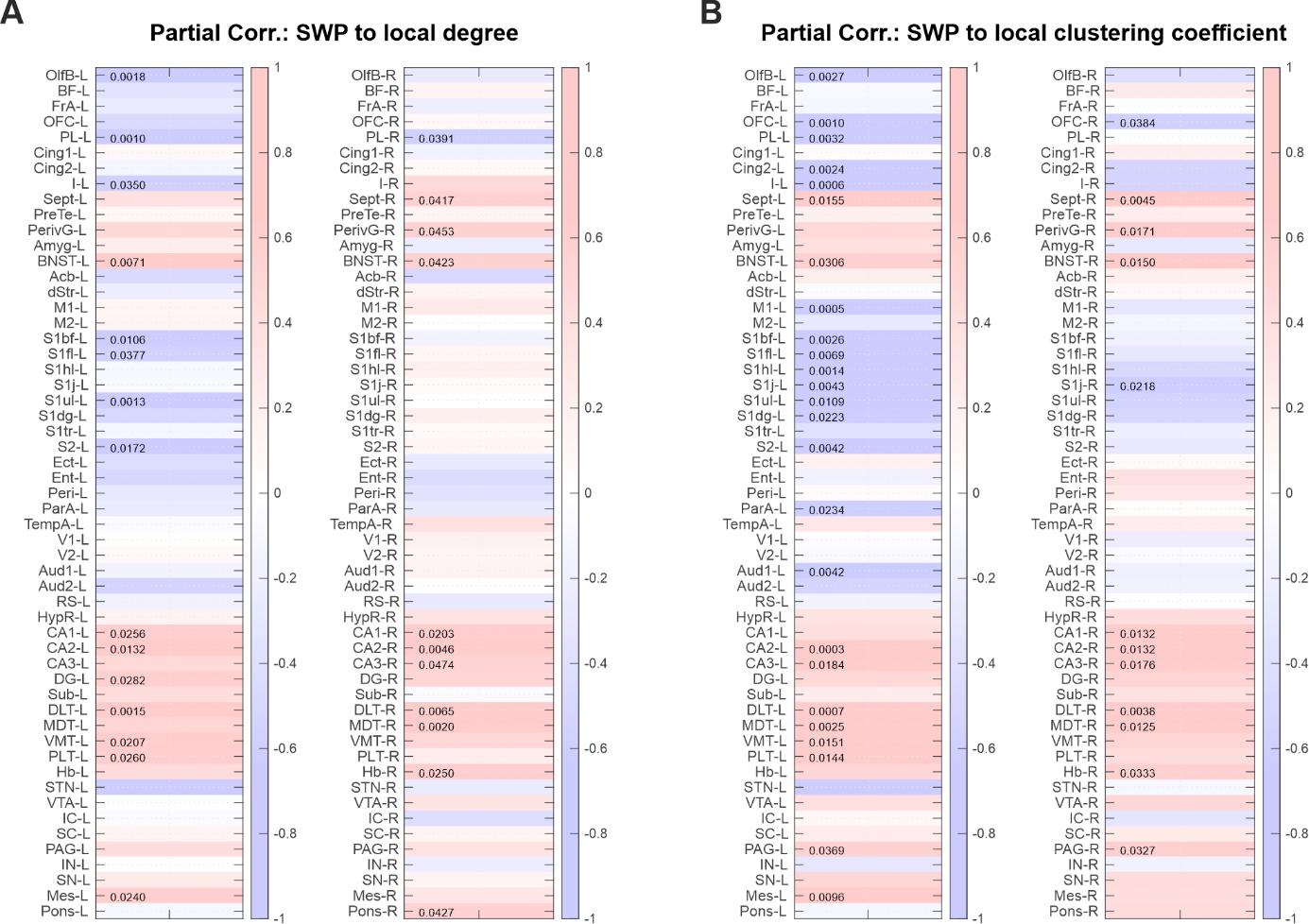


Figure S10. **Partial correlation coefficients between small-world propensity (SWP) and local graph metrics (local degree and clustering coefficient) for 55 unilateral brain regions (with isoflurane level as covariate) in DAT-KO rats. (A)** The DAT-KO-specific partial correlation coefficients between SWP and local degree. **(B)** The DAT-KO-specific partial correlation coefficients between SWP and local clustering coefficient. While partial correlation is mostly positive for subcortical hippocampal and thalamic regions (red), prefrontal and somato-motor cortical areas demonstrate predominantly negative correlations to SWP (blue). Hence, the general disturbance of whole-brain organization quantified by decreased SWP in DAT-KO rats compared to WT rats (see **Figure 4** of the main paper) is largely driven by frontal-midbrain decoupling.

Significant p-values are denoted in the respective boxes of the plot.

L-R, left-right hemispheres. Abbreviations for brain regions are the same as in **Figure S4**.

**REFERENCES**

1. Frischknecht U, Hermann D, Tunc-Skarka N, Wang GY, Sack M, van Eijk J, et al. Negative association between MR-spectroscopic glutamate markers and gray matter volume after alcohol withdrawal in the hippocampus: A translational study in humans and rats. Alcohol Clin Exp Res 2017; 41: 323-33.

2. Gass N, Becker R, Reinwald J, Cosa-Linan A, Sack M, Weber-Fahr W, et al. The influence of ketamine's repeated treatment on brain topology does not suggest an antidepressant efficacy. Transl Psychiatry 2020; 10: 56,020-0727-8.

3. Reinwald JR, Sartorius A, Weber-Fahr W, Sack M, Becker R, Didriksen M, et al. Separable neural mechanisms for the pleiotropic association of copy number variants with neuropsychiatric traits. Transl Psychiatry 2020; 10: 93,020-0771-4.

4. Schneider M, Spanagel R. Appetitive odor-cue conditioning attenuates the acoustic startle response in rats. Behav Brain Res 2008; 189: 226-30.

5. Barriere DA, Magalhaes R, Novais A, Marques P, Selingue E, Geffroy F, et al. The SIGMA rat brain templates and atlases for multimodal MRI data analysis and visualization. Nat Commun 2019; 10: 5699,019-13575-7.

6. Chou N, Wu J, Bai Bingren J, Qiu A, Chuang KH. Robust automatic rodent brain extraction using 3-D pulse-coupled neural networks (PCNN). IEEE Trans Image Process 2011; 20: 2554-64.

7. Rubinov M, Sporns O. Complex network measures of brain connectivity: Uses and interpretations. Neuroimage 2010; 52: 1059-69.

8. Watts DJ, Strogatz SH. Collective dynamics of 'small-world' networks. Nature 1998; 393: 440-2.

9. Newman ME. Finding community structure in networks using the eigenvectors of matrices. Phys Rev E Stat Nonlin Soft Matter Phys 2006; 74: 036104.

10. Schneider CM, Moreira AA, Andrade JS Jr, Havlin S, Herrmann HJ. Mitigation of malicious attacks on networks. Proc Natl Acad Sci U S A 2011; 108: 3838-41.

11. Muldoon SF, Bridgeford EW, Bassett DS. Small-world propensity and weighted brain networks. Sci Rep 2016; 6: 22057.

12. Afyouni S, Nichols TE. Insight and inference for DVARS. Neuroimage 2018; 172: 291-312.
